# The clinical and microbiological characteristics of enteric fever in Cambodia, 2008-2015

**DOI:** 10.1371/journal.pntd.0005964

**Published:** 2017-09-20

**Authors:** Laura M. F. Kuijpers, Thong Phe, Chhun H. Veng, Kruy Lim, Sovann Ieng, Chun Kham, Nizar Fawal, Laetitia Fabre, Simon Le Hello, Erika Vlieghe, François-Xavier Weill, Jan Jacobs, Willy E. Peetermans

**Affiliations:** 1 Department of Clinical Sciences, Institute of Tropical Medicine, Antwerp, Belgium; 2 Department of Microbiology & Immunology, KU Leuven, Leuven, Belgium; 3 Sihanouk Hospital Center of HOPE, Phnom Penh, Cambodia; 4 Unité des Bactéries Pathogènes Entériques, Centre National de Référence des *E*. *coli*, *Shigella* et *Salmonella*, Institut Pasteur, Paris, France; 5 Department of General Internal Medicine, Infectious diseases and Tropical Medicine, University Hospital Antwerp, Antwerp, Belgium; 6 Department of Internal Medicine, University Hospital Leuven, Leuven, Belgium; Oxford University Clinical Research Unit Vietnam, VIET NAM

## Abstract

**Background:**

Enteric fever remains a major public health problem in low resource settings and antibiotic resistance is increasing. In Asia, an increasing proportion of infections is caused by *Salmonella enterica* serovar Paratyphi A, which for a long time was assumed to cause a milder clinical syndrome compared to *Salmonella enterica* serovar Typhi.

**Methodology:**

A retrospective chart review study was conducted of 254 unique cases of blood culture confirmed enteric fever who presented at a referral adult hospital in Phnom Penh, Cambodia between 2008 and 2015. Demographic, clinical and laboratory data were collected from clinical charts and antibiotic susceptibility testing was performed. Whole genome sequence analysis was performed on a subset of 121 isolates.

**Results:**

One-hundred-and-ninety unique patients were diagnosed with *Salmonella* Paratyphi A and 64 with *Salmonella* Typhi. In the period 2008–2012, *Salmonella* Paratyphi A comprised 25.5% of 47 enteric fever cases compared to 86.0% of 207 cases during 2013–2015. Presenting symptoms were identical for both serovars but higher median leukocyte counts (6.8 x 10^9^/L vs. 6.3 x 10^9^/L; *p* = 0.035) and C-reactive protein (CRP) values (47.0 mg/L vs. 36 mg/L; *p* = 0.034) were observed for *Salmonella* Typhi infections. All but one of the *Salmonella* Typhi isolates belonged to haplotype H58 associated with multidrug resistance (MDR) (*i*.*e*. resistance to ampicillin, chloramphenicol and co-trimoxazole).;42.9% actually displayed MDR compared to none of the *Salmonella* Paratyphi A isolates. Decreased ciprofloxacin susceptibility (DCS) was observed in 96.9% (62/64) of *Salmonella* Typhi isolates versus 11.5% (21/183) of *Salmonella* Paratyphi A isolates (all but one from 2015). All isolates were susceptible to azithromycin and ceftriaxone.

**Conclusions:**

In Phnom Penh, Cambodia, *Salmonella* Paratyphi A now causes the majority of enteric fever cases and decreased susceptibility against ciprofloxacin is increasing. Overall, *Salmonella* Typhi was significantly more associated with MDR and DCS compared to *Salmonella* Paratyphi A.

## Author summary

Enteric fever is a bloodstream infection caused by the bacteria *Salmonella* Typhi or *Salmonella* Paratyphi A, B, or C. It is common in low resource settings and linked to poor water quality and sanitation. The disease is also endemic in Cambodia and since 2013 there has been a sharp increase in the number of *Salmonella* Paratyphi A infections. We sought to compare the clinical phenotypes and antibiotic susceptibilities of *Salmonella* Paratyphi A infections with those of *Salmonella* Typhi infections in this setting. We retrospectively collected demographic, clinical and laboratory data from clinical charts of 254 patients with a blood culture positive for enteric fever. We also assessed antibiotic susceptibility patterns and sequenced the genome of a subset of isolates. We found that since 2013 the majority of enteric fever cases are caused by *Salmonella* Paratyphi A which increasingly shows decreased susceptibility to the antibiotic ciprofloxacin, the current first line treatment. In contrast, in *Salmonella* Typhi a re-emergence of susceptibility for the former first line antibiotics of ampicillin, co-trimoxazole and chloramphenicol was observed. Presenting symptoms of *Salmonella* Typhi and *Salmonella* Paratyphi A were identical, minor differences were observed in laboratory parameters.

## Introduction

*Salmonella enterica* serovar Typhi (*Salmonella* Typhi) and *Salmonella enterica* serovars Paratyphi (*Salmonella* Paratyphi) A, B, and C are Gram-negative bacteria which can invade the bloodstream and cause typhoid and paratyphoid fever respectively (also jointly known as ‘enteric fever’). They are confined to the human host and are transmitted via the fecal-oral route. Enteric fever poses a serious disease burden in low resource settings where the infection is linked to poor sanitation and limited access to safe drinking water [1]. Although enteric fever has become rare in Western countries it continues to affect international travelers returning from endemic countries [2].

Patients with enteric fever typically present with acute fever and non-specific symptoms. For a long time, *Salmonella* Paratyphi A was thought to cause milder disease than *Salmonella* Typhi but several studies have contradicted this [1–4].

For both serovars, antibiotic resistance is increasingly reported and there is now widespread presence of co-resistance against the former first line treatment options of ampicillin, co-trimoxazole and chloramphenicol (known as ‘multidrug resistance’) and decreased susceptibility to ciprofloxacin, the current first line drug [5, 6]. Resistance to ciprofloxacin is also increasingly reported [7, 8]. Worrisome are recent reports on emerging resistance against third-generation cephalosporins and azithromycin, the current alternative treatment options [9, 10].

Although historically the majority of enteric fever cases were caused by *Salmonella* Typhi, the proportion of *Salmonella* Paratyphi A infections has been increasing steadily since the turn of the century, in particular on the Asian continent [11].

In 2013, a significant increase in *Salmonella* Paratyphi A infections was also observed in Cambodia, a country where enteric fever remains one of the most common clinical and blood culture-confirmed diseases [12]. The increase was described in local residents as well as in travelers returning from Cambodia to Europe, New Zealand, Japan and the United States [13–16].

Surprisingly, little is known about the clinical and microbiological characteristics of *Salmonella* Typhi and *Salmonella* Paratyphi A infections in Cambodian adults.

This study therefore aims to assess the clinical and microbiological aspects of enteric fever in patients attending an adult hospital in Phnom Penh, Cambodia, during 2008–2015. More specifically it aims to assess differences between infections caused by *Salmonella* Typhi as compared to *Salmonella* Paratyphi A.

## Materials and methods

### Study setting & population

Sihanouk Hospital Center of HOPE (SHCH) is a 40-bed non-governmental referral hospital for adults in Phnom Penh, Cambodia. Since July 2007, SHCH and the Institute of Tropical Medicine (ITM) in Antwerp, Belgium, have been jointly organising the surveillance of bloodstream infections at this hospital and its associated clinics. For this study, all data collected between 2008–2015 were analyzed. Blood cultures were systematically sampled in all patients presenting at SHCH who were suspected of having sepsis according to the Systemic Inflammatory Response Syndrome (SIRS) criteria [17]. Recently, new definitions and criteria for sepsis have been proposed such as the Sequential [Sepsis-related] Organ Failure Assessment (SOFA) score [18]. Over the 8-year period, 18,927 blood cultures were sampled from mostly, but not exclusively, adults [19]. Of these cultures 1,654 (8,7%) yielded clinically significant organisms.

### Clinical review

From all patients whose blood was drawn for culture, basic demographic and clinical data were registered in a surveillance logbook. A medical doctor verified missing data with patients during a routine phone call one week after discharge from the hospital which was part of standard care. In addition, for this study, the available medical charts of all patients with blood culture-confirmed enteric fever were reviewed retrospectively by a second medical doctor for additional symptoms and signs.

### Laboratory methods

Hematology parameters were analyzed using a Sysmex KX-21 and T-1800i analyzer (Sysmex Corporation, Kobe, Japan) and CRP values were measured using a TEMIS Linear Analyzer (Linear Chemicals sl, Montgat, Spain).

Blood cultures were sampled and worked-up as previously described [20]. Isolates biochemically identified as *Salmonella* spp. were stored at -70°C on porous beads in cryopreservative (Microbank, Pro-Lab Diagnostics, Richmond Hill, Canada) and eventually shipped to the ITM in Belgium. At ITM, the isolates were serotyped using commercial antisera (Sifin, Berlin, Germany) following the White-Kauffmann-Le Minor scheme. A selection of 91 isolates were sent to the Institut Pasteur in Paris for confirmation and whole genome sequencing.

At the ITM, antibiotic susceptibility was determined for all available isolates by disk diffusion on Mueller-Hinton II agar in accordance with the CLSI 2016 guidelines [21]. The following antimicrobial drugs (Neo-Sensitabs, Rosco, Taastrup, Denmark) were tested: ampicillin, sulfamethoxazole-trimethoprim, chloramphenicol, nalidixic acid, pefloxacin, gentamicin, tetracycline, ceftriaxone, ceftazidime, meropenem and ertapenem. Nalidixic acid and pefloxacin served as predictors for ciprofloxacin non-susceptibility.

In addition, for all available isolates, minimal inhibitory concentration (MIC) values for ciprofloxacin and azithromycin were determined by the E-test macro method (bioMérieux, Marcy L'Etoile, France).

Quality control was performed using *Escherichia coli* (ATCC 25922) and *Staphylococcus aureus* (ATCC 29213).

Multidrug resistance (MDR) was defined as co-resistance to ampicillin, chloramphenicol and trimethoprim-sulfamethoxazole [22]. For comparison with previously published literature, we used the superseded term ‘decreased ciprofloxacin susceptibility (DCS)’, defined as MIC-values of ≥0.12 mg/L and ≤0.5 mg/L, *i*.*e*. currently classified as ‘intermediate susceptibility' but associated with treatment failures or delayed treatment response [21].

### Assessment of antimicrobial resistance genes and *Salmonella* Typhi H58 typing

Whole genome sequencing was carried out on all 65 *Salmonella* Typhi isolates and a selection of 26 *Salmonella* Paratyphi A isolates at the Plateforme de microbiologie mutualisée (P2M) from the Pasteur International Bioresources network (PIBnet, Institut Pasteur, Paris, France). Short-read sequences from 30 previously published *Salmonella* Paratyphi A genomes were also included [23]. The run accession numbers and related metadata are detailed in S1 Table. Short-read sequences have been deposited to the European Nucleotide Archive (ENA) (http://www.ebi.ac.uk/ena) (accession number PRJEB19906).

The MagNAPure 96 system (Roche Diagnostics, Indianopolis, IN, USA) was used for DNA extraction, libraries were prepared using the Nextera XT kit (Illumina, San Diego, CA, USA) and sequencing was done with the NextSeq 500 system (Illumina). Read alignment, single nucleotide polymorphism (SNP) detection and maximum-likelihood phylogeny were carried out as described previously [23]. Sequence assembly was performed using SPAdes v. 3.6.0 [24].

*Salmonella* Typhi isolates were categorized as belonging to haplotype H58 based on the presence of the H58 specific single SNP (T at nucleotide 252 on the gene *glpA* corresponding to STY2513 from GenBank accession no. AL513382, *Salmonella* Typhi CT18) [25]. Genotyphi (https://github.com/katholt/genotyphi) was also used to classify *Salmonella* Typhi [26]. *Salmonella* Paratyphi A isolates were categorized as belonging to clade C5 (the dominant clade in Cambodia) based on the presence of the C5-specific SNP (G to A at position 2 381 607 within the SPA_RS11495 gene) [23]. The presence of antibiotic resistance genes was determined with ResFinder version 2.1 (https://cge.cbs.dtu.dk/services/ResFinder/) [27] and plasmids with PlasmidFinder version 1.3 (https://cge.cbs.dtu.dk/services/PlasmidFinder/) and pMLST version 1.4 (https://cge.cbs.dtu.dk/services/pMLST-1.4/) [27, 28]. The presence of mutations in the quinolone-resistance determining region of the DNA gyrase and topoisomerase IV genes (*gyrA*, *gyrB*, *parC* and *parE*) was assessed by the visual examination of sequences.

### Data registration, statistical analysis

Demographic, clinical and microbiological data were entered encoded into an Excel database that was created for this study (Microsoft, Redmond, WA, USA). The code referring to the patient identity was only known by two medical doctors. Access to the database was restricted to these two medical doctors and patient identifiers were removed prior to analysis.

Only the first isolate and associated clinical data for each unique patient was considered. Isolates recovered from a second blood culture drawn within two weeks after the initial one were considered as duplicates, whereas isolates recovered from a repeat blood culture more than two weeks after the initial one were considered as recurrences (either relapse or repeat infections). Statistical analysis was done with Stata 12 (Stata Corp., College Station, TX, USA). Continuous variables are described by a median and interquartile range (IQR). Comparisons between *Salmonella* Typhi and *Salmonella* Paratyphi were performed using a Mann-Whitney U test for continuous values and a Chi square test or Fisher exact test for proportions. A *p*-value of < 0.05 was considered significant.

### Ethics

The study was conducted according to the principles expressed in the Declaration of Helsinki and involves use of information that was previously collected in the course of routine care. Ethical approval for the Microbiological Surveillance Study was granted by the Institutional Review Board of the ITM, the Ethics Committee of Antwerp University, and the National Ethics Committee for Health Research in Cambodia. This study and approval includes retrospective review of demographic and clinical data which are part of routine clinical history taking as recorded in the clinical chart.

## Results

### Surveillance of invasive *Salmonella* infections

Between 1 January 2008 and 31 December 2015 193 *Salmonella* Paratyphi A isolates were retrieved from 190 patients and 65 *Salmonella* Typhi isolates from 64 patients. There were no *Salmonella* Paratyphi B or C isolates; sixty-two non-typhoidal *Salmonella* isolates were retrieved from 49 patients. The combined annual proportion of *Salmonella* Typhi and *Salmonella* Paratyphi A among all clinically significant organisms varied between 2.8% (2008) and 31.7% (2014).

**Fig 1:**
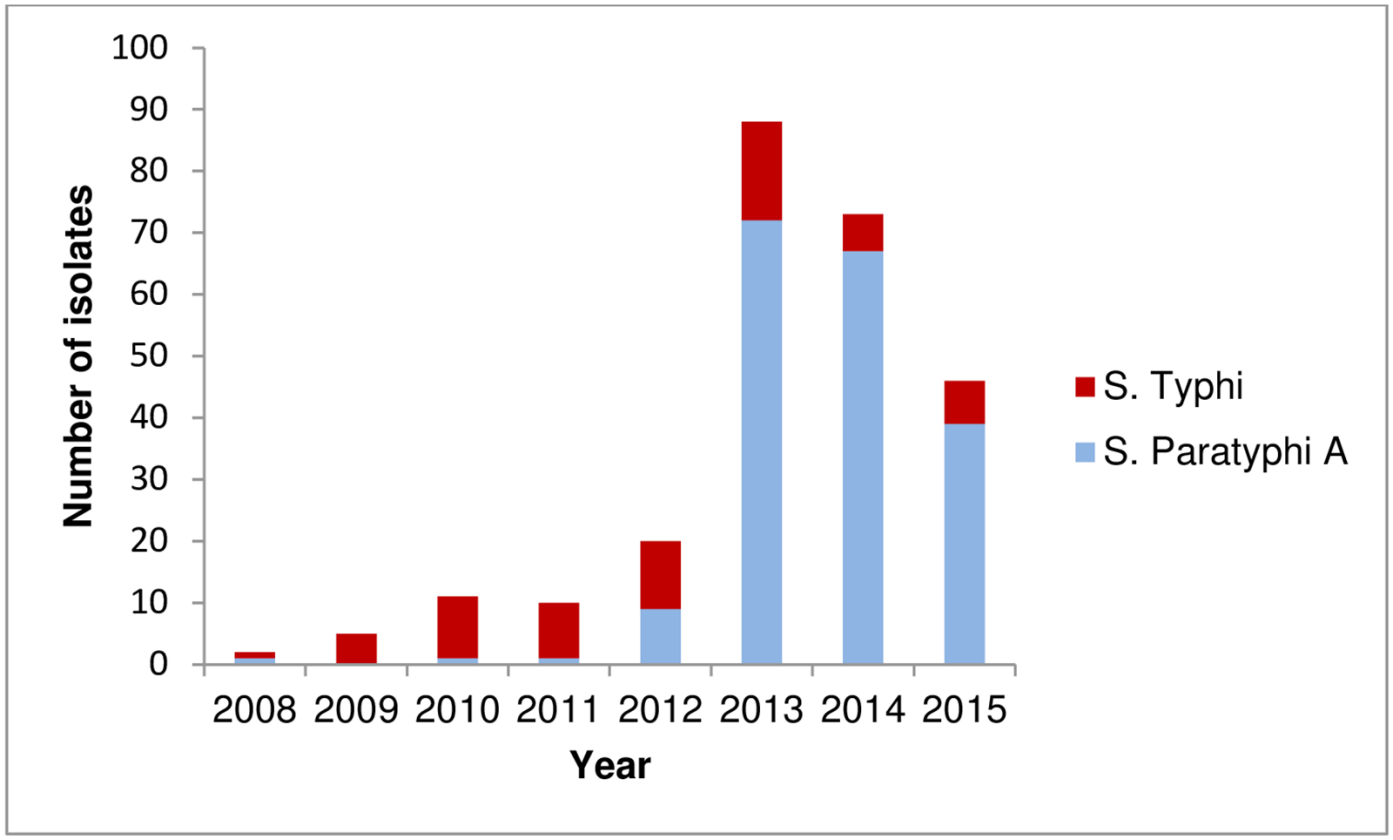
Number of unique confirmed blood-culture *Salmonella enterica* serovar Paratyphi A and *Salmonella enterica* serovar Typhi isolates at the Sihanouk Hospital Center of HOPE, Phnom Penh, Cambodia, 2008–2015.

During 2008–2012, enteric fever was caused mostly by *Salmonella* Typhi (35 cases) and only 12 cases of *Salmonella* Paratyphi A infection were identified (Fig 1). In 2013 however, a sharp increase in the number of *Salmonella* Paratyphi A cases was observed with a total of 72 unique cases. In 2014 and 2015, the absolute annual number of *Salmonella* Paratyphi A cases decreased, but remained higher than for the period preceding 2013. During this period, the number of *Salmonella* Typhi cases remained relatively stable.

**Fig 2:**
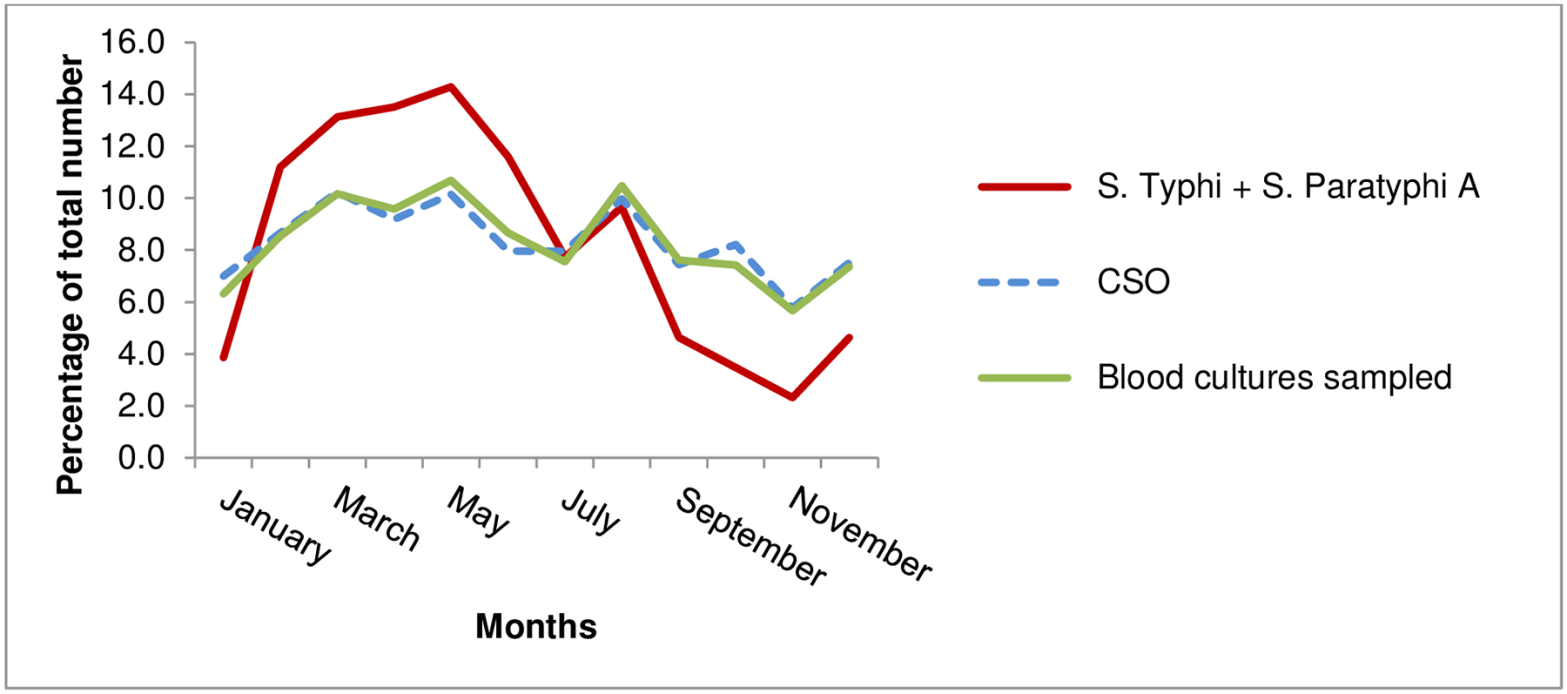
Cumulative monthly percentages of total number of blood culture bottles sampled, clinically significant organisms (CSO) and *Salmonella* Paratyphi A and *Salmonella* Typhi isolated from blood cultures at the Sihanouk Hospital Center of HOPE, Phnom Penh, Cambodia.

The majority of *Salmonella* Paratyphi A (64.7%; 123/190) and *Salmonella* Typhi infections (59.4%; 38/64) cases occurred during the dry season (months November—April) while there was an overall decreasing trend during the rainy season (months June-October) (Fig 2). Compared to the monthly percentage of total blood cultures sampled and clinically significant organisms found, the monthly combined percentage of *Salmonella* Typhi and *Salmonella* Paratyphi A was higher during the hot and dry season (March—May) and lower during the rainy season (June-October).

### Recurrent infections

There were four cases of recurrent infections (37–48 days interval between first and recurrent infection), three with *Salmonella* Paratyphi A and one with *Salmonella* Typhi; whole genome sequence data was available for three of the four pairs. SNP analysis of the paired isolates revealed that they differed by only two or three SNPs and the isolate pairs formed discrete clusters within the trees (S1 and S2 Figs).

### Epidemiological and clinical features

Available epidemiological, clinical and radiographic findings of all enteric fever patients are listed in Table 1.

**Table 1:** Epidemiological, clinical and radiographic findings for patients presenting with enteric fever, according to pathogen (*Salmonella* Typhi versus *Salmonella* Paratyphi A).

	N	*Salmonella* Typhi	N	*Salmonella* Paratyphi A	Significant *p*-values
*Epidemiology (numbers (% of total))*					
Male	64	32 (50)	190	93 (48.9)	
Phnom Penh resident	63	38 (60.3)	189	154 (81.5)	0.001
Hospitalization	63	11 (17.5)	188	19 (10.1)	
Previous antibiotic exposure^a^					
*Epidemiology (median (IQR))*					
Median age, years	64	23 (18–30)	190	26 (22–31)	0.015
Median duration of symptoms, days	40	4 (3–7)	112	4 (3–7)	
Median duration of hospitalization, days	10^b^	5.5 (3–6)	19	4 (2–5)	
*Symptom and signs (numbers (% of total))*					
Fever	48	48 (100)	182	181 (99.5)	
Abdominal pain	48	30 (62.5)	182	113 (62.1)	
Diarrhea	48	12 (25.0)	182	40 (22.0)	
Constipation	48	4 (8.3)	182	10 (5.5)	
Vomiting	48	11 (22.9)	182	27 (14.8)	
Headache	43	24 (55.8)	178	114 (64.0)	
Cough	40	15 (37.5)	168	46 (27.4)	
*Physical findings (median (IQR))*					
Temperature, °C	46	38.4 (37.3–39.0)	179	38.0 (37.0–38.8)	
Pulse, beats/min.	46	106 (95–119)	179	105 (91–117)	
Systolic blood pressure, mm Hg	46	107 (100–117)	179	113 (103–123)	0.036
Diastolic blood pressure, mm Hg	46	67.5 (60–77)	179	70 (64–78)	
*Physical findings (numbers (% of total))*					
Fever (Temperature >38°C)	46	26 (56.5)	179	88 (49.2)	
Tachycardia (Pulse >100 beats/min)^c^	46	27 (58.7)	179	109 (60.9)	
Abdominal tenderness	39	15 (38.5)	164	56 (34.1)	
*Radiographic signs*					
Ultrasound: Splenomegaly	11	3 (27.3)	38	5 (13.2)	
Ultrasound: Hepatomegaly	11	0 (0)	38	6 (15.8)	
Chest X-ray: Normal	10	9 (90.0)	32	31 (96.9)	

For statistical analysis the X2 test or Fisher exact test was used for proportions and the Mann-Whitney U test for continuous variables. Only statistically significant p-values are listed

IQR = Interquartile range

^a^ = Two weeks before presentation

^b^ = Data on duration of hospitalization was missing for one *Salmonella* Typhi patient

^c^ = For children aged 5–12 years (N = 4) tachycardia was defined as >120 beats/min

Eleven out of 189 (5.8%) *Salmonella* Paratyphi A patients had a known co-morbidity, *i*.*e*. HIV (*n* = 7), Diabetes Mellitus type 1 or 2 (*n* = 3) and leukemia (*n* = 1). Of 63 *Salmonella* Typhi patients also nine (14.3%) had a known co-morbidity, *i*.*e*. HIV (*n* = 7) and Diabetes Mellitus type 1 (*n* = 2). All but one HIV patient were on antiretroviral therapy at time of presentation. There were 11 patients known to have hepatitis B-positive serology (8 *Salmonella* Paratyphi A, 3 *Salmonella* Typhi). At least five could be classified as inactive chronic carriers and two had signs of chronic liver disease on ultrasound.

Patients with a *Salmonella* Paratyphi A infection were more likely to be living in Phnom Penh compared to *Salmonella* Typhi patients (81.5% (154/189) vs. 60.3% (38/63); *p* = 0.001) but the median duration of illness at presentation was the same (four days).

Eleven (17.5%) patients with typhoid fever and 19 (10.1%) with paratyphoid fever were hospitalized, with no statistically significant difference between the two groups (*p* = 0.119). Reasons for hospitalization included sepsis, persistent fever despite antibiotic therapy, dizziness due to low blood pressure, suspicion of dengue hemorrhagic fever (thrombocytopenia) and dysregulated diabetes mellitus. There were no deaths nor complications noted.

The most frequently reported symptoms in all enteric fever patients together were fever in 229 patients (99.6%), headache in 138 (62.4%) and abdominal pain in 143 (62,2%). Presence or absence of classic enteric fever signs such as a coated tongue and rose spots were infrequently mentioned in clinical files and therefore not evaluated.

Despite the non-specific symptoms, physicians noted typhoid fever in their differential diagnosis upon admission in 67.7% (136/201) of the cases.

There were no statistically significant differences in individual symptoms between typhoid and paratyphoid fever patients, but patients infected with *Salmonella* Typhi had a slightly but significantly lower median systolic blood pressure (107 mm Hg vs. 113 mm Hg; *p* = 0.036).

Treatment was not systematically recorded for all patients as many were lost to follow-up. Various antimicrobial regimens were used, but ceftriaxone (2g I.V., once daily) was given most frequently as empirical treatment and as monotherapy, normally for 10–14 days. In case of de-escalation to oral antibiotics, this concerned mostly ciprofloxacin (500 mg, twice daily) and next amoxicillin/clavulanate (625 mg, three times a day). In case of persistent fever while awaiting blood culture results, amikacin was occasionally added to ceftriaxone.

### Laboratory parameters

The laboratory parameters of enteric fever patients on admission are summarized in Table 2. Common laboratory abnormalities for enteric fever patients included moderately risen transaminase levels in 133 patients (70.7%), an elevated CRP in 53 patients (94.6%) and eosinopenia in 49 patients (90.7%). Hematological abnormalities were uncommon; the leukocyte count was normal in 88.1% of all patients. Compared to *Salmonella* Paratyphi A infected patients, *Salmonella* Typhi patients had slightly but significantly higher median values for leukocytes (6.8 x 10^9^/L vs. 6.3 x 10^9^/L; *p* = 0.035) and C-reactive protein (CRP) (47.0 mg/L vs. 36 mg/L; *p* = 0.034), with more presence of leukocytosis (10.0% vs. 2.2% *p* = 0.015). *Salmonella* Paratyphi A infection was associated with a higher monocyte count compared to *Salmonella* Typhi (0.48 x 10^9^/L vs. 0.33 x 10^9^/L), but this difference did not reach statistical significance (*p* = 0.069).

**Table 2:** Laboratory parameters at presentation for enteric fever patients attending the Sihanouk Hospital Center of HOPE in Phnom Penh, Cambodia.

	N	*Salmonella* Typhi	N	*Salmonella* Paratyphi A	*Significant p*-values
*Values expressed as median (IQR)*					
Hemoglobin, g/L	60	13.1 (12.1–14.2)	186	13.2 (12.2–14.5)	
Leukocyte count, cells x 10^9^/L	60	6.8 (5.6–8.8)	186	6.3 (5.1–7.6)	0.035
Monocyte count, cells x 10^9^/L	12	0.33 (0.28–0.55)	41	0.48 (0.37–0.72)	
Eosinophil count, cells x 10^9^/L	12	0 (0–0.01)	41	0 (0–0.03)	
Basophil count, cells x 10^9^/L	12	0.02 (0.01–0.03)	40^b^	0.01 (0.01–0.02)	
Thrombocyte count, cells x 10^9^/L	52	205 (166–251)	185	205 (165–247)	
CRP, mg/L	13	47.0 (38.6–79.2)	43	36.0 (25.2–54.7)	0.034
AST, units/L	40	47.0 (33.5–81.5)	148	43.0 (30.5–72.5)	
ALT, units/L	40	38.5 (26–88)	148	39.0 (24.5–72.5)	
Sodium mmol/L	33	132 (130–135)	105	135 (132–137)	0.024
Potassium mmol/L	33	3.9 (3.7–4.2)	105	3.9 (3.6–4.1)	
Chloride mmol/L	32	99 (96–101)	105	100 (97–102)	
*Values expressed as numbers (% of total)*					
Leukocytosis (leukocytes >11 x10^9^/L)	60	6 (10)	186	4 (2.2)	0.015
Leukopenia (leukocytes <4x10^9^/L)	60	4 (6.7)	186	17 (9.1)	
Eosinopenia (eosinophils <0.08x10^9^/L)	13	13 (100)	41	36 (87.8)	
Thrombocytopenia (<150x10^9^L)	52	11 (21.2)	185	31 (16.8)	
Elevated AST and/or ALT (units/L)^a^	40	28 (70.0)	148	105 (70.9)	

For statistical analysis the X2 test or Fisher exact test was used for proportions and the Mann-Whitney U test for continuous variables. Only statistically significant p-values are listed

ALT = Alanine transaminase

AST = Aspartate transaminase

CRP = C-reactive protein

IQR = Interquartile range

^a^ = ≥ 18 years: Males: AST ≥35U/L and ALT ≥45U/L; Females: AST ≥31U/L and ALT ≥34U/L; 13–17 years: AST ≥42U/L and ALT ≥45U/L; 7–12 years: AST ≥48U/L and ALT ≥44U/L

^b^ = Basophil count not measurable in 1 patient

### Microbiological features

Both anaerobic and aerobic blood cultures showed signs of growth after a median of two days (IQR 2–3) for all enteric fever patients. In 221 enteric fever patients a pair of one aerobic bottle and one anaerobic bottle was sampled, and in 180 of those cases (81.4%) both bottles grew. In the other cases (growth in only a single bottle), it was the aerobic bottle which grew in nearly two-thirds (65.9%; 27/41) of pairs.

Reported antibiotic exposure in the two weeks before blood culture sampling was not associated with a difference in the median days to growth for both aerobic bottles and anaerobic bottles.

In total, 183 out of 190 (96.3%) unique *Salmonella* Paratyphi A isolates and all 64 unique *Salmonella* Typhi isolates recovered during the study period were available for antibiotic susceptibility testing (Table 3).

**Table 3:** Antibiotic resistance rates of 64 *Salmonella* Typhi isolates and 183 *Salmonella* Paratyphi A isolates, Sihanouk Hospital Center of HOPE, Phnom Penh, Cambodia.

	2008–2012	2013–2015	2008–2012	2013–2015
	*Salmonella* Typhi (N = 35) % resistant	*Salmonella* Typhi (N = 29) % resistant	*Salmonella* Paratyphi A (N = 12) % resistant	*Salmonella* Paratyphi A (N = 171) % resistant
MDR	62.9	17.2	0	0
Nalidixic acid	97.1^a^	93.1	0	12.3
DCS	100	93.1	0	12.3
MDR + DCS	62.9	17.2	0	0
Tetracycline	62.9	24.1	0	0
Gentamicin	0	0	0	0
Ceftriaxone	0	0	0	0
Azithromycin	0	0	0	0
Meropenem	0	0	0	0
Ertapenem	0	0	0	0

MDR = Multidrug resistance

DCS = Decreased ciprofloxacin susceptibility. Defined as MIC value ≥0.12 mg/L and ≤0.5 mg/L

^a^ = The remaining isolate displayed intermediate resistance to nalidixic acid

For *Salmonella* Typhi, there was a significant decrease (*p* = <0.001) in the proportion of isolates that were MDR over the 8-year period (62.9% vs. 17.2%) while decreased susceptibility to ciprofloxacin remained at nearly 100% (96.9%; 62/64) during the entire period. For *Salmonella* Paratyphi A the emergence of DCS was noted as of 2015 (S1 Table). In this year 19 out of 36 unique isolates (52.8%) showed DCS.

Overall, *Salmonella* Typhi was significantly more likely to be MDR and more likely to display DCS than was *Salmonella* Paratyphi A (42.2% vs. 0.0%; *p* = <0.001 and 96.9% vs. 11.5%; *p* = <0.001 respectively).

Of note, for both serovars no ciprofloxacin resistance was reported and the presence of nalidixic acid and pefloxacin resistance were excellent predictors of DCS except in case of one isolate with a single *gyrB* mutation (Table 4 and S1 Table). Furthermore, no resistance against third-generation cephalosporins, carbapenems or azithromycin was found.

**Table 4:** Mutations found in the DNA gyrase genes in 62 *Salmonella enterica* serovar Typhi isolates and 21 *Salmonella enterica* serovar Paratyphi A isolates.

Serovar	Resistance phenotype	MIC-value ciprofloxacin (mg/L)	*gyrA*	*gyrB*
*Salmonella* Typhi (n = 62)	NaR, PefR, DCS	0.19–0.38	Ser83Phe (n = 60)	-
	NaR, PefR, DCS	0.38	Asp87Asn + Ser83Phe (n = 1)	-
	NaI, PefR, DCS	0.094	-	Ser464Phe (n = 1)
*Salmonella* Paratyphi A (n = 21)	NaR, PefR, DCS	0.25–0.38	Ser83Phe (n = 20)	-
	NaR, PefR, DCS	0.125	Asp87Gly (n = 1)	-

NaR = Nalidixic acid resistant. NaI = Nalidixic acid intermediate susceptible. PefR = Pefloxacin resistant. DCS = Decreased Ciprofloxacin Susceptibility. MIC = Minimum Inhibitory Concentration.

### Molecular analysis of subtypes and resistance mechanisms

All but one of the *Salmonella* Typhi isolates were confirmed to be of the H58 haplotype (recently reclassified as the 4.3.1 genotype), with only 27 out of 63 (42.9%) unique H58 isolates displaying MDR but 98.4% (62/63) displaying DCS. The only non-H58 isolate belonged to genotype 3.2.1 and was pan-susceptible. All of the *Salmonella* Paratyphi A isolates with DCS belonged to the C5 clade.

Most frequently observed in both *Salmonella* Typhi and *Salmonella* Paratyphi A with DCS was the *gyrA* mutation leading to serine-to-phenylalanine substitution at codon 83 (Ser83Phe) (Table 4). These isolates showed DCS and resistance to pefloxacin and nalidixic acid. There was one *Salmonella* Typhi isolate with a double *gyrA* mutation and one with a *gyrB* mutation. The latter mutation (serine-to-phenylalanine substitution at codon 464 (Ser464Phe)) was associated with intermediate susceptibility to nalidixic acid and DCS. No mutations in *ParC or ParE* were observed.

Various resistance genes (*bla*_TEM-1B_, *catA1*, *sul1*, *sul2*, *dfrA7*, *tet(B)*, *strAB*) were detected in MDR *Salmonella* Typhi which were associated with the presence of an incHI1 PST6 plasmid (S1 Table).

## Discussion

The present study describes the clinical and microbiological aspects of enteric fever in a large group of patients attending an adult hospital in Cambodia with several interesting results.

First, and in line with some other Asian countries, a clear increase was noted in the proportion of *Salmonella* Paratyphi A infections. This can largely be explained by a community outbreak which occurred in 2013, but the number of cases has remained high also in succeeding years. A recent genetic study on the Cambodian *Salmonella* Paratyphi A outbreak isolates showed that these isolates belong to a clade that has been circulating in the South-East Asian region already for decades [23]. Further, no indications were found for significant genetic changes within the Cambodian isolates suggesting that environmental and/or behavioral factors are more likely to play a role.

Patients with paratyphoid fever were significantly more likely than typhoid fever patients to be residents of Phnom Penh, which suggests that exposure to the bacterium is more common in the city. Previous studies from Nepal and Indonesia have linked paratyphoid fever to recent immigration into the capital and consumption of street food [29, 30]. Increased dependency on street food has been linked to urbanization, and Phnom Penh is rapidly expanding.

As part of urban expansion, some of the city’s peri-urban lakes have been filled with sand to reclaim land [31]. These lakes are estimated to receive 80% of the city’s (untreated) waste water and act as a natural sewage treatment through aquatic cultivation of vegetables of which some are consumed raw [32]. Reductions in the size of these lakes could have led to higher concentrations of fecal sludge and bacteria in the remaining water and increased flooding in the city [33].

The majority of enteric fever cases occurred in the dry season. During this season more vegetables are harvested and it is also known for an increased availability of snails and clams (bivalve shellfish), due to low water levels in rivers. Shellfish are known to be able to concentrate micro-organisms from water. They are popular snacks which are dried outside rather than boiled during the dry season. In addition, this season coincides with the two most important festivities of the year, the Chinese and Khmer New Year which are associated with increased migration in and out of the city and longer storage duration of food. Last, high daily temperatures may lead to more indiscriminate intake of water and ice cubes. These factors are currently being explored more in-depth.

Second, based on individual symptoms at presentation, infections caused by *Salmonella* Typhi *vs*. *Salmonella* Paratyphi A were similar and indistinguishable, which is in line with other studies from Asia [3, 4]. For both serovars, the median pulse rates at presentation (106 and 105 beats/minute) were high. This has been noted before in children with enteric fever [34].

No complications or deaths occurred which could be ascribed to a prompt start of antibiotic therapy and an early presentation. The latter can also explain the absence of relative bradycardia and the low rate of diarrhea observed which are typically seen in later stages of the disease [35].

In general, laboratory abnormalities were non-specific and leukocyte counts were normal in 87.4% of all enteric fever patients which was also found by others [36]. Higher leukocyte counts and CRP values were found among *Salmonella* Typhi-infected patients, suggesting a more severe infection. This is in line with a recent human challenge study, which found that a challenge with *Salmonella* Paratyphi A in healthy volunteers resulted in a milder disease profile (high rates of afebrile bacteremia) than that observed following typhoid challenge [37].

Some differences in presentation were noted when comparing these results to travelers infected with the same *Salmonella* Paratyphi A C5 strain returning from Cambodia to France. In the latter study, the majority of patients did have diarrhea (70.6%) and were hospitalized (86%) [14]. This difference may be due to other waterborne or oral-fecal infections travelers frequently contract and/or less financial constrains related to hospital admission [2]. Clinical presentation in the present study also differed from typhoid fever patients in African countries where higher rates of severe complications and mortality are observed [38]. As the same H58 haplotype of *Salmonella* Typhi is dominant in Asia and in eastern Africa, differences could perhaps be explained by timely access to health care and adequate treatment as well as to host-related factors including underlying co-morbidities like malnutrition.

It has been suggested that isolates of the H58 lineage and MDR strains in general are associated with increased virulence and pathogenicity [39–41]. Therefore the results regarding the clinical presentation and severity of cases as described here, might not be applicable to areas where other lineages dominate.

As a third observation, antibiotic resistance trends were very different for the two serovars. While 42.2% of the unique *Salmonella* Typhi isolates displayed MDR, none of the *Salmonella* Paratyphi A isolates did. DCS was present in nearly all *Salmonella* Typhi isolates, but only emerged in *Salmonella* Paratyphi A from 2015. The rapid increase of DCS in *Salmonella* Paratyphi during that year is of concern as ciprofloxacin is the treatment of choice for uncomplicated enteric fever.

Fourth, although all but one *Salmonella* Typhi isolates were found to belong to the globally dominant H58 haplotype, more than half were not associated with MDR and the proportion of (plasmid-mediated) MDR *Salmonella* Typhi significantly decreased during the study period. This trend has previously been noted in India, Nepal and neighboring country Vietnam [42–44], but contrasts with a recent study on *Salmonella* Typhi isolates from rural Cambodia where 89% of the H58 isolates displayed the MDR phenotype [45]. The re-emergence of susceptibility might result from a lack of antibiotic pressure since fluoroquinolones have become the preferred treatment both in community and hospital settings in Phnom Penh.

No resistance against ceftriaxone nor against azithromycin was observed. However, reports on extended spectrum beta-lactamases (ESBL) positive and azithromycin resistant *Salmonella* spp. isolates are emerging globally including one from the same hospital on *Salmonella enterica* serovar Choleraesuis [20, 46, 47] underlining the importance of continued microbiological surveillance.

Last, molecular analysis of isolates from three patients with a recurrent infection showed relapse was more likely than re-infection with isolate pairs differing only 2–3 SNPs which can occur during the period of persistence within the human body and suggests relapse rather than re-infection [48]. Relapse is estimated to occur in around 5–10% of enteric fever cases usually two to three weeks after the resolution of fever [5]. In our study, a blood culture confirmed recurrence was witnessed only in 4 out of 254 cases (1.6%). It is likely that other recurrent infections have been missed, partly due to the different medical systems that co-exist in Cambodia in which patients readily switch from one healthcare provider to another, especially if symptoms persist.

This study has several limitations. First, the hospital-based setting precluded generalization to patients whose symptoms were not severe enough to seek medical care in a hospital or clinic. Second, the study concerned mostly adults and therefore findings might not be equally applicable to a pediatric population. Third, the study was retrospective in nature; not all clinical charts were available for review and clinical record keeping was variable among different clinicians. It was not possible to reliably estimate time to defervescence.

Despite these limitations, this is one of the largest and most comprehensive descriptive studies on *Salmonella* Paratyphi A infections so far which is relevant given the global increase in *Salmonella* Paratyphi A infections. The data do not represent one single hospital, but several clinics located in different districts of the city. Some of these clinics have reduced rates for the poor and during the study period all blood cultures were provided for free. This helped to overcome some of the bias associated with a hospital based study.

The high proportion of *Salmonella* Typhi and Paratyphi A recovered from blood cultures indicated that enteric fever is a very frequent disease in Phnom Penh. While efforts are made to increase the microbiological diagnostic capacity in the country, a rapid test for invasive *Salmonella* infections would be a welcome tool for fast and reliable diagnosis. It could increase knowledge on the burden of disease in the community and could replace the flawed Widal test that is still frequently used.

As the current *Salmonella* Typhi vaccine provides no to very little protection against *Salmonella* Paratyphi A, the development of an effective *Salmonella* Paratyphi A vaccine should be promoted, pending improved water quality and sanitation [49].

## Conclusion

Enteric fever is frequent in Phnom Penh and the proportion of cases due to *Salmonella* Paratyphi A has increased. Studies to investigate risk factors and possible transmission routes are urgently needed to advise public health interventions. No MDR was observed for *Salmonella* Paratyphi A but DCS increased rapidly. DCS remained highly prevalent in *Salmonella* Typhi while MDR rates have declined. Ceftriaxone and azithromycin remain highly active in vitro but continued surveillance is imperative to monitor resistance.

## Supporting information

S1 Table Strain list and accession numbers for organisms used in this study. (XLSX)

S1 Fig Maximum Likelihood (ML) tree of 185 *Salmonella* Paratyphi A genomes. Maximum Likelihood (ML) tree of 185 *Salmonella* Paratyphi A genomes including 159 previously published genomes (Kuijpers & Le Hello et al., 2016 [23] and Zhou et al., 2014 [50]). Fifty-six genomes represent isolates collected at the Sihanouk Hospital Center of HOPE, Phnom Penh, Cambodia between 2008–2015. For readability, only the position of the reference genome (*Salmonella* Paratyphi A ATCC 9150) and the paired isolates are shown. Only clades C1-C5 are indicated. The big arrow indicates the paired isolates (ID 6778 and 6748, 2 SNPs difference; ID 6610 and 6670; 3 SNPs difference).
(PPTX)

S2 Fig Maximum Likelihood (ML) tree of 66 *Salmonella* Typhi genomes. Maximum Likelihood (ML) tree of 66 *Salmonella* Typhi genomes including the *Salmonella* Typhi CT18 reference genome (AL513382) and 65 genomes of *Salmonella* Typhi isolates collected at the Sihanouk Hospital Center of HOPE, Phnom Penh, Cambodia between 2008–2015. For readability, only the position of the reference genome (*Salmonella* Typhi CT18) and of the paired isolates (ID 4764 and 4855, 3 SNPs difference) are indicated (with a big arrow).
(PPTX)

S1 Checklist STROBE checklist. (PDF)

## References

[1] Bhan MK, Bahl R, Bhatnagar S. Typhoid and paratyphoid fever. Lancet. 2005;366(9487):749–62. doi: 10.1016/S0140-6736(05)67181-4 16125594 10.1016/S0140-6736(05)67181-4

[2] Meltzer E, Sadik C, Schwartz E. Enteric fever in Israeli travelers: a nationwide study. J Travel Med. 2005;12(5):275–81. 16256052 10.2310/7060.2005.12507

[3] Maskey AP, Day JN, Phung QT, Thwaites GE, Campbell JI, Zimmerman M, et al *Salmonella enterica* serovar Paratyphi A and S. enterica serovar Typhi cause indistinguishable clinical syndromes in Kathmandu, Nepal. Clin Infect Dis. 2006;42(9):1247–53. doi: 10.1086/503033 16586383 10.1086/503033

[4] Vollaard AM, Ali S, Widjaja S, Asten HA, Visser LG, Surjadi C, et al Identification of typhoid fever and paratyphoid fever cases at presentation in outpatient clinics in Jakarta, Indonesia. Trans R Soc Trop Med Hyg. 2005;99(6):440–50. doi: 10.1016/j.trstmh.2004.09.012 15837356 10.1016/j.trstmh.2004.09.012

[5] Parry CM, Hien TT, Dougan G, White NJ, Farrar JJ. Typhoid fever. N Engl J Med. 2002;347(22):1770–82. doi: 10.1056/NEJMra020201 12456854 10.1056/NEJMra020201

[6] Wain J, Kidgell C. The emergence of multidrug resistance to antimicrobial agents for the treatment of typhoid fever. Trans R Soc Trop Med Hyg. 2004;98(7):423–30. doi: 10.1016/j.trstmh.2003.10.015 15138079 10.1016/j.trstmh.2003.10.015

[7] Medalla F, Sjolund-Karlsson M, Shin S, Harvey E, Joyce K, Theobald L, et al Ciprofloxacin-resistant *Salmonella enterica* Serotype Typhi, United States, 1999–2008. Emerg Infect Dis. 2011;17(6):1095–8. doi: 10.3201/eid/1706.100594 21749779 10.3201/eid1706.100594 PMC3363319

[8] Chiou CS, Lauderdale TL, Phung DC, Watanabe H, Kuo JC, Wang PJ, et al Antimicrobial resistance in *Salmonella enterica* Serovar Typhi isolates from Bangladesh, Indonesia, Taiwan, and Vietnam. Antimicrob Agents Chemother. 2014;58(11):6501–7. doi: 10.1128/AAC.03608-14 25136011 10.1128/AAC.03608-14 PMC4249406

[9] Pokharel BM, Koirala J, Dahal RK, Mishra SK, Khadga PK, Tuladhar NR. Multidrug-resistant and extended-spectrum beta-lactamase (ESBL)-producing *Salmonella enterica* (serotypes Typhi and Paratyphi A) from blood isolates in Nepal: surveillance of resistance and a search for newer alternatives. Int J Infect Dis. 2006;10(6):434–8. doi: 10.1016/j.ijid.2006.07.001 16978898 10.1016/j.ijid.2006.07.001

[10] Hassing RJ, Goessens WH, van Pelt W, Mevius DJ, Stricker BH, Molhoek N, et al *Salmonella* subtypes with increased MICs for azithromycin in travelers returned to The Netherlands. Emerg Infect Dis. 2014;20(4):705–8. doi: 10.3201/eid2004.131536 24655478 10.3201/eid2004.131536 PMC3966360

[11] Ochiai RL, Wang X, von Seidlein L, Yang J, Bhutta ZA, Bhattacharya SK, et al *Salmonella* paratyphi A rates, Asia. Emerg Infect Dis. 2005;11(11):1764–6. doi: 10.3201/eid1111.050168 16318734 10.3201/eid1111.050168 PMC3367370

[12] Vlieghe ER, Phe T, De Smet B, Veng HC, Kham C, Lim K, et al Bloodstream infection among adults in Phnom Penh, Cambodia: key pathogens and resistance patterns. PLoS One. 2013;8(3):e59775 doi: 10.1371/journal.pone.0059775 23555777 10.1371/journal.pone.0059775 PMC3612098

[13] Vlieghe E, Phe T, De Smet B, Veng CH, Kham C, Sar D, et al Increase in *Salmonella enterica* serovar Paratyphi A infections in Phnom Penh, Cambodia, January 2011 to August 2013. Euro Surveill. 2013;18(39). 10.2807/1560-7917.es2013.18.39.20592 24094060

[14] Tourdjman M, Le Hello S, Gossner C, Delmas G, Tubiana S, Fabre L, et al Unusual increase in reported cases of paratyphoid A fever among travellers returning from Cambodia, January to September 2013. Euro Surveill. 2013;18(39). 10.2807/1560-7917.es2013.18.39.20594 24094059

[15] Saitoh T, Morita M, Shimada T, Izumiya H, Kanayama A, Oishi K, et al Increase in paratyphoid fever cases in Japanese travellers returning from Cambodia in 2013. Epidemiol Infect. 2016;144(3):602–6. doi: 10.1017/S0950268815001648 26169980 10.1017/S0950268815001648

[16] Judd MC, Grass JE, Mintz ED, Bicknese A, Mahon BE. *Salmonella enterica* Paratyphi A Infections in Travelers Returning from Cambodia, United States. Emerg Infect Dis. 2015;21(6):1089–91. doi: 10.3201/eid2106.150088 25988984 10.3201/eid2106.150088 PMC4451907

[17] Bone RC, Balk RA, Cerra FB, Dellinger RP, Fein AM, Knaus WA, et al Definitions for sepsis and organ failure and guidelines for the use of innovative therapies in sepsis. The ACCP/SCCM Consensus Conference Committee. American College of Chest Physicians/Society of Critical Care Medicine. Chest. 1992;101(6):1644–55. 1303622 10.1378/chest.101.6.1644

[18] Seymour CW, Liu VX, Iwashyna TJ, Brunkhorst FM, Rea TD, Scherag A, et al Assessment of Clinical Criteria for Sepsis: For the Third International Consensus Definitions for Sepsis and Septic Shock (Sepsis-3). JAMA. 2016;315(8):762–74. doi: 10.1001/jama.2016.0288 26903335 10.1001/jama.2016.0288 PMC5433435

[19] Phe T VE, Lim K, Veng CH, Thai S, Leng L, Kham C, Jacobs J. Surveillance of bloodstream infection and antibiotic resistance in Phnom Penh, Cambodia (2007–2014). Poster presented at: 17th International Congress on Infectious Diseases; 2016 March 2–5; Hyderabad, India.

[20] Vlieghe ER, Phe T, De Smet B, Veng CH, Kham C, Bertrand S, et al Azithromycin and ciprofloxacin resistance in *Salmonella* bloodstream infections in Cambodian adults. PLoS Negl Trop Dis. 2012;6(12):e1933 doi: 10.1371/journal.pntd.0001933 23272255 10.1371/journal.pntd.0001933 PMC3521708

[21] Clinical and Laboratory Standards Institute (CLSI). Performance standards for antimicrobial susceptibility testing. 26th informational supplement. CLSI Document M100-S26. Wayne, PA: CLSI 2016.

[22] World Health Organization (WHO). Background document: the diagnosis, treatment and prevention of typhoid fever. 2003.

[23] Kuijpers LMF, Le Hello S, Fawal N, Fabre L, Tourdjman M, Dufour M, Sar D, Kham C, Phe T, Vlieghe E, Bouchier C, Jacobs J, Weill FX. Genomic analysis of *Salmonella enterica* serotype Paratyphi A during an outbreak in Cambodia, 2013–2015. Microbial Genomics. 2016; doi: 10.1099/mgen.0.000092 28348832 10.1099/mgen.0.000092 PMC5320704

[24] Bankevich A, Nurk S, Antipov D, Gurevich AA, Dvorkin M, Kulikov AS, et al SPAdes: a new genome assembly algorithm and its applications to single-cell sequencing. J Comput Biol. 2012;19(5):455–77. doi: 10.1089/cmb.2012.0021 22506599 10.1089/cmb.2012.0021 PMC3342519

[25] Roumagnac P, Weill FX, Dolecek C, Baker S, Brisse S, Chinh NT, et al Evolutionary history of *Salmonella* typhi. Science. 2006;314(5803):1301–4. doi: 10.1126/science.1134933 17124322 10.1126/science.1134933 PMC2652035

[26] Wong VK, Baker S, Connor TR, Pickard D, Page AJ, Dave J, et al An extended genotyping framework for *Salmonella enterica* serovar Typhi, the cause of human typhoid. Nat Commun. 2016;7:12827 doi: 10.1038/ncomms12827 27703135 10.1038/ncomms12827 PMC5059462

[27] Zankari E, Hasman H, Cosentino S, Vestergaard M, Rasmussen S, Lund O, et al Identification of acquired antimicrobial resistance genes. J Antimicrob Chemother. 2012;67(11):2640–4. doi: 10.1093/jac/dks261 22782487 10.1093/jac/dks261 PMC3468078

[28] Carattoli A, Zankari E, Garcia-Fernandez A, Voldby Larsen M, Lund O, Villa L, et al In silico detection and typing of plasmids using PlasmidFinder and plasmid multilocus sequence typing. Antimicrob Agents Chemother. 2014;58(7):3895–903. doi: 10.1128/AAC.02412-14 24777092 10.1128/AAC.02412-14 PMC4068535

[29] Vollaard AM, Ali S, van Asten HA, Widjaja S, Visser LG, Surjadi C, et al Risk factors for typhoid and paratyphoid fever in Jakarta, Indonesia. JAMA. 2004;291(21):2607–15. doi: 10.1001/jama.291.21.2607 15173152 10.1001/jama.291.21.2607

[30] Karkey A, Thompson CN, Tran Vu Thieu N, Dongol S, Le Thi Phuong T, Voong Vinh P, et al Differential epidemiology of *Salmonella* Typhi and Paratyphi A in Kathmandu, Nepal: a matched case control investigation in a highly endemic enteric fever setting. PLoS Negl Trop Dis. 2013;7(8):e2391 doi: 10.1371/journal.pntd.0002391 23991240 10.1371/journal.pntd.0002391 PMC3749961

[31] Halim H, Muong, V. Capital’s remaining freshwater lake a sinking ship. Phnom Penh Post. 2017 Feb 23. http://www.phnompenhpost.com/post-property/capitals-remaining-freshwater-lake-sinking-ship

[32] Sar S, Chervier C, Lim P, Warrender C, Warrender GW, Gilbert RG. Seasonal Direct-Use Value of Cheung Ek Peri-Urban Lake, Phnom Penh, Cambodia. Int J Environmental & Rural Development. 2010;1:113–8

[33] Yeap C. Faecal build-up a threat: study. The Phnom Penh Post. 2012 Apr 25. http://www.phnompenhpost.com/national/faecal-build-threat-study

[34] Davis TM, Makepeace AE, Dallimore EA, Choo KE. Relative bradycardia is not a feature of enteric fever in children. Clin Infect Dis. 1999;28(3):582–6. doi: 10.1086/515143 10194082 10.1086/515143

[35] Cunha BA. Osler on typhoid fever: differentiating typhoid from typhus and malaria. Infect Dis Clin North Am. 2004;18(1):111–25. doi: 10.1016/S0891-5520(03)00094-1 15081508 10.1016/S0891-5520(03)00094-1

[36] Caumes E, Ehya N, Nguyen J, Bricaire F. Typhoid and paratyphoid fever: a 10-year retrospective study of 41 cases in a Parisian hospital. J Travel Med. 2001;8(6):293–7. 11726293 10.2310/7060.2001.22378

[37] Dobinson HC, Gibani MM, Jones C, Thomaides-Brears HB, Voysey M, Darton TC, et al Evaluation of the Clinical and Microbiological Response to *Salmonella* Paratyphi A Infection in the First Paratyphoid Human Challenge Model. Clin Infect Dis. 2017;64(8):1066–73. doi: 10.1093/cid/cix042 28158395 10.1093/cid/cix042 PMC5439345

[38] Otegbayo JA, Daramola OO, Onyegbutulem HC, Balogun WF, Oguntoye OO. Retrospective analysis of typhoid fever in a tropical tertiary health facility. Trop Gastroenterol. 2002;23(1):9–12. 12170927

[39] Wong VK, Baker S, Pickard DJ, Parkhill J, Page AJ, Feasey NA, et al Phylogeographical analysis of the dominant multidrug-resistant H58 clade of *Salmonella* Typhi identifies inter- and intracontinental transmission events. Nat Genet. 2015;47(6):632–9. doi: 10.1038/ng.3281 25961941 10.1038/ng.3281 PMC4921243

[40] Bhutta ZA. Impact of age and drug resistance on mortality in typhoid fever. Arch Dis Child. 1996;75(3):214–7. 8976660 10.1136/adc.75.3.214 PMC1511710

[41] Wain J, Pham VB, Ha V, Nguyen NM, To SD, Walsh AL, et al Quantitation of bacteria in bone marrow from patients with typhoid fever: relationship between counts and clinical features. J Clin Microbiol. 2001;39(4):1571–6. doi: 10.1128/JCM.39.4.1571-1576.2001 11283089 10.1128/JCM.39.4.1571-1576.2001 PMC87972

[42] Shrestha KL, Pant ND, Bhandari R, Khatri S, Shrestha B, Lekhak B. Re-emergence of the susceptibility of the *Salmonella* spp. isolated from blood samples to conventional first line antibiotics. Antimicrob Resist Infect Control. 2016;5:22 doi: 10.1186/s13756-016-0121-8 27231547 10.1186/s13756-016-0121-8 PMC4881163

[43] Menezes GA, Harish BN, Khan MA, Goessens WH, Hays JP. Antimicrobial resistance trends in blood culture positive *Salmonella* Typhi isolates from Pondicherry, India, 2005–2009. Clin Microbiol Infect. 2012;18(3):239–45. doi: 10.1111/j.1469-0691.2011.03546.x 21714829 10.1111/j.1469-0691.2011.03546.x

[44] Le TA, Fabre L, Roumagnac P, Grimont PA, Scavizzi MR, Weill FX. Clonal expansion and microevolution of quinolone-resistant *Salmonella enterica* serotype typhi in Vietnam from 1996 to 2004. J Clin Microbiol. 2007;45(11):3485–92. doi: 10.1128/JCM.00948-07 17728470 10.1128/JCM.00948-07 PMC2168509

[45] Pham Thanh D, Thompson CN, Rabaa MA, Sona S, Sopheary S, Kumar V, et al The Molecular and Spatial Epidemiology of Typhoid Fever in Rural Cambodia. PLoS Negl Trop Dis. 2016;10(6):e0004785 doi: 10.1371/journal.pntd.0004785 27331909 10.1371/journal.pntd.0004785 PMC4917249

[46] Nair S, Ashton P, Doumith M, Connell S, Painset A, Mwaigwisya S, et al WGS for surveillance of antimicrobial resistance: a pilot study to detect the prevalence and mechanism of resistance to azithromycin in a UK population of non-typhoidal *Salmonella*. J Antimicrob Chemother. 2016;71(12):3400–3408. doi: 10.1093/jac/dkw318 27585964 10.1093/jac/dkw318

[47] Kalonji LM, Post A, Phoba MF, Falay D, Ngbonda D, Muyembe JJ, et al Invasive *Salmonella* Infections at Multiple Surveillance Sites in the Democratic Republic of the Congo, 2011–2014. Clin Infect Dis. 2015;61 Suppl 4:S346–53. 26449951 10.1093/cid/civ713

[48] Okoro CK, Kingsley RA, Quail MA, Kankwatira AM, Feasey NA, Parkhill J, et al High-resolution single nucleotide polymorphism analysis distinguishes recrudescence and reinfection in recurrent invasive nontyphoidal *Salmonella* typhimurium disease. Clin Infect Dis. 2012;54(7):955–63. doi: 10.1093/cid/cir1032 22318974 10.1093/cid/cir1032 PMC3297646

[49] Simanjuntak CH, Paleologo FP, Punjabi NH, Darmowigoto R, Soeprawoto, Totosudirjo H, et al Oral immunisation against typhoid fever in Indonesia with Ty21a vaccine. Lancet. 1991;338(8774):1055–9. 1681365 10.1016/0140-6736(91)91910-m

[50] Zhou Z, McCann A, Weill FX, Blin C, Nair S, Wain J, et al Transient Darwinian selection in *Salmonella enterica* serovar Paratyphi A during 450 years of global spread of enteric fever. Proc Natl Acad Sci U S A. 2014;111(33):12199–204. doi: 10.1073/pnas.1411012111 25092320 10.1073/pnas.1411012111 PMC4143038

