# Supplementary figures and images for: The clinical and microbiological characteristics of enteric fever in Cambodia, 2008-2015
Source: PLoS Negl Trop Dis. 2017 Sep 20;11(9):e0005964. doi: 10.1371/journal.pntd.0005964 (PMC5624643; doi:10.1371/journal.pntd.0005964)

## Slide 1
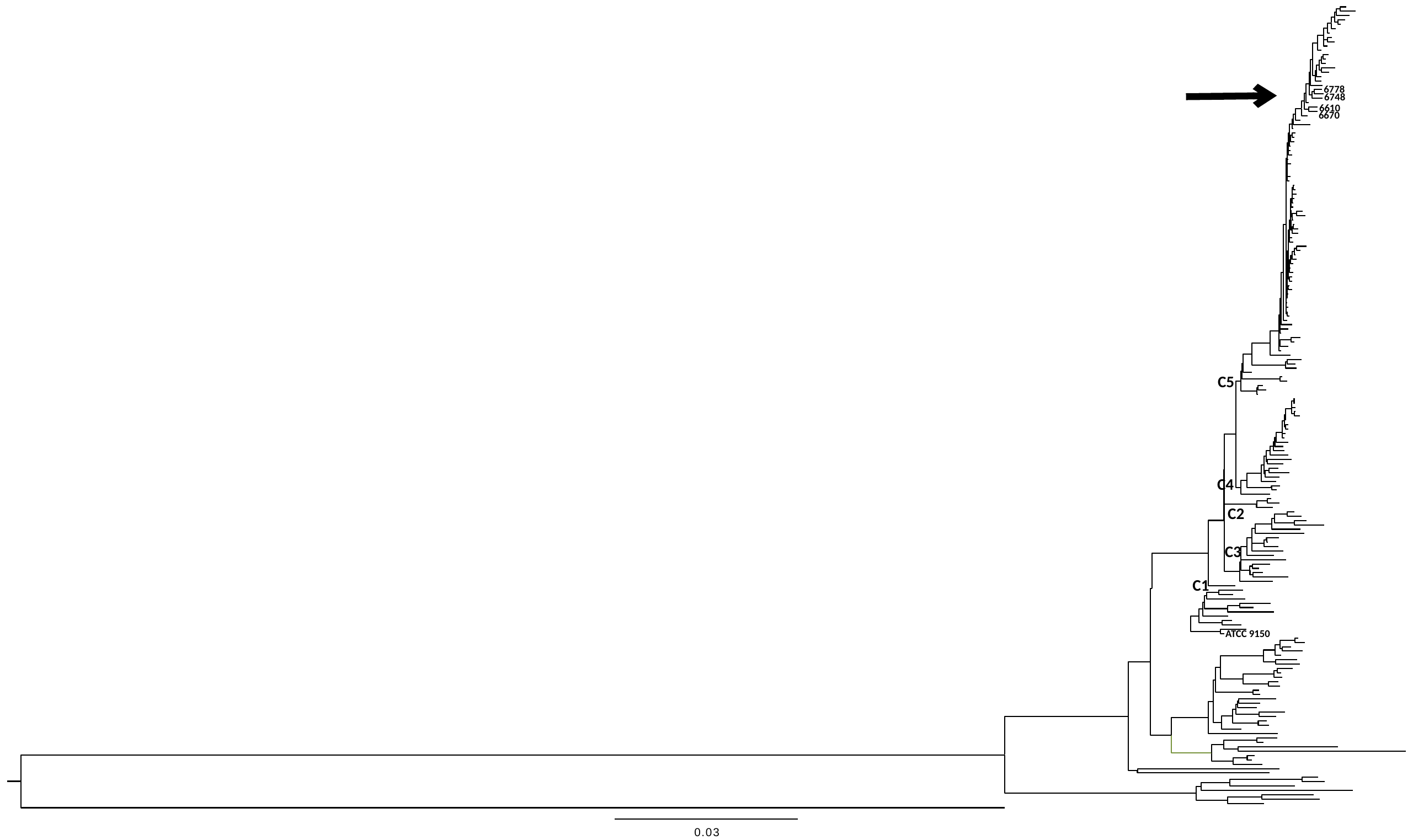

6778
6748
6610
6670
C5
C4
C2
C3
C1
ATCC 9150
0.03

Supplement: S1 Fig — Maximum Likelihood (ML) tree of 185 Salmonella Paratyphi A genomes including 159 previously published genomes (Kuijpers & Le Hello et al., 2016 [23] and Zhou et al., 2014 [50]). Fifty-six genomes represent isolates collected at the Sihanouk Hospital Center of HOPE, Phnom Penh, Cambodia between 2008–2015. For readability, only the position of the reference genome (Salmonella Paratyphi A ATCC 9150) and the paired isolates are shown. Only clades C1-C5 are indicated. The big arrow indicates the paired isolates (ID 6778 and 6748, 2 SNPs difference; ID 6610 and 6670; 3 SNPs difference). (PPTX) [file pntd.0005964.s002.pptx]

## Slide 1
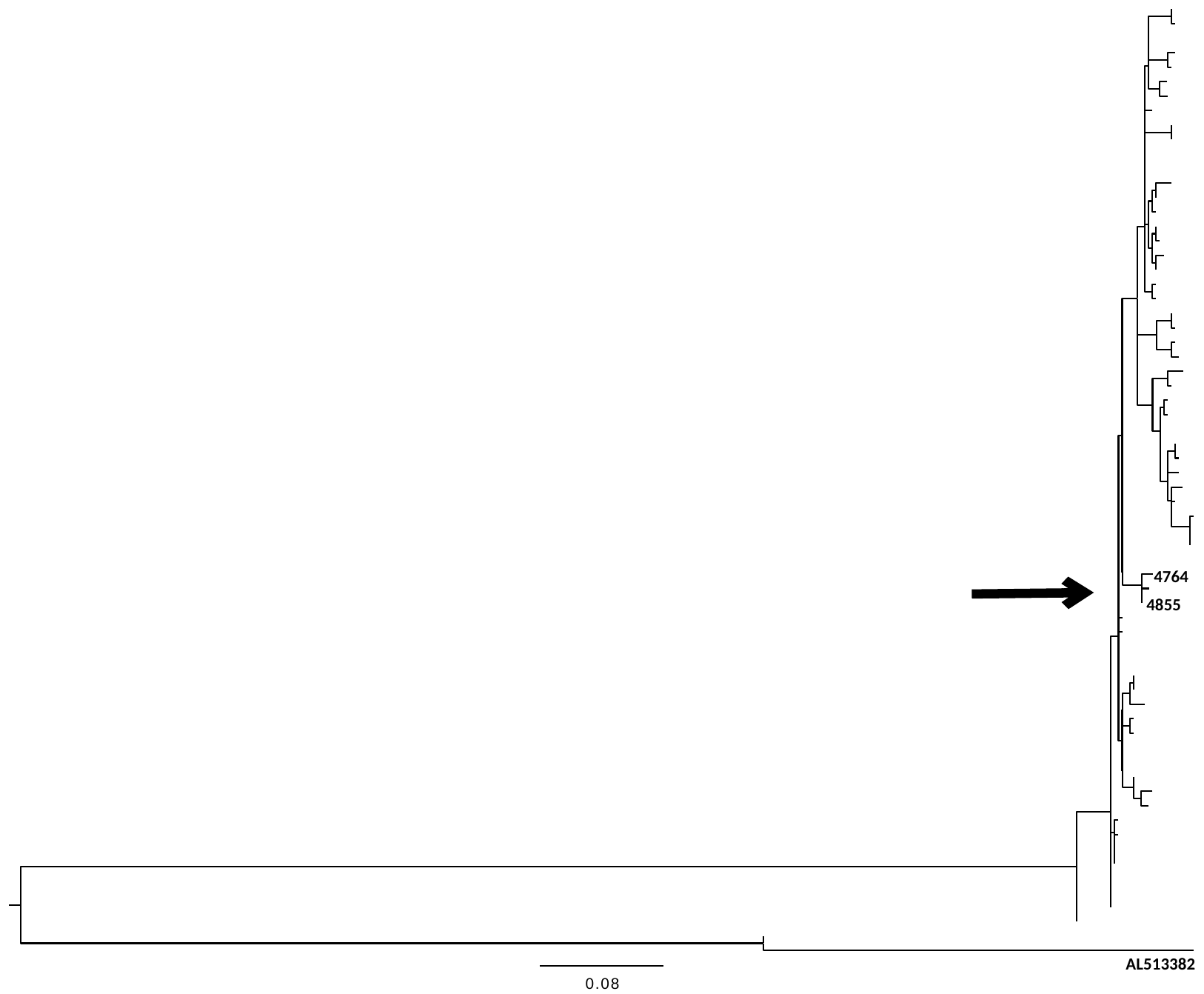

4764
4855
AL513382
0.08

Supplement: S2 Fig — Maximum Likelihood (ML) tree of 66 Salmonella Typhi genomes including the Salmonella Typhi CT18 reference genome (AL513382) and 65 genomes of Salmonella Typhi isolates collected at the Sihanouk Hospital Center of HOPE, Phnom Penh, Cambodia between 2008–2015. For readability, only the position of the reference genome (Salmonella Typhi CT18) and of the paired isolates (ID 4764 and 4855, 3 SNPs difference) are indicated (with a big arrow). (PPTX) [file pntd.0005964.s003.pptx]
